# Polyphenols Content and In Vitro α-Glycosidase Activity of Different Italian Monofloral Honeys, and Their Effect on Selected Pathogenic and Probiotic Bacteria

**DOI:** 10.3390/microorganisms9081694

**Published:** 2021-08-09

**Authors:** Florinda Fratianni, Maria Neve Ombra, Antonio d’Acierno, Lucia Caputo, Giuseppe Amato, Vincenzo De Feo, Raffaele Coppola, Filomena Nazzaro

**Affiliations:** 1 Institute of Food Science, CNR-ISA, Via Roma 64, 83100 Avellino, Italy; fratianni@isa.cnr.it (F.F.); nombra@isa.cnr.it (M.N.O.); dacierno.a@isa.cnr.it (A.d.); 2 Department of Pharmacy, University of Salerno, via Giovanni Paolo II, 84084 Fisciano, Italy; lcaputo@unisa.it (L.C.); g.amato29@studenti.unisa.it (G.A.); 3 Department of Agricultural, Environmental and Food Sciences, University of Molise, Via de Sanctis snc, 86100 Campobasso, Italy; coppola@unimol.it

**Keywords:** honey, polyphenols, α-glycosidase, biofilm, probiotics, prebiotics

## Abstract

We evaluated the polyphenol content and the α-glucosidase activity exhibited by different monofloral honeys of Italian origin. Their capacity to act on different pathogenic (*Acinetobacter baumannii*, *Escherichia coli*, *Listeria monocytogenes*, *Pseudomonas aeruginosa*, and *Staphylococcus aureus)* as well as probiotic bacteria (*Lacticaseibacillus casei*, *Lactobacillus* *acidophilus*, *Lactiplantibacillus plantarum*, *Lactobacillus gasseri*, and *Lacticaseibacillus rhamnosus*) was also assessed. Total polyphenols varied between 110.46 μg/g of fresh product (rhododendron honey) and 552.29 μg/g of fresh product (strawberry tree honey). Such result did not correspond to a parallel inhibitory α-glycosidase activity that, in each case was never higher than 33 μg/mL. Honeys were differently capable to fight the biofilm formation of the pathogens (inhibition up to 93.27%); they inhibited the in vitro adhesive process (inhibition up to 84.27%), and acted on mature biofilm (with values up to 76.64%). Their effect on bacterial metabolism was different too. Honeys were ineffective to inhibit *E. coli* mature biofilm nor to act on its metabolism. The action of the honey on probiotic strains seemed almost always stimulate their growth. Thus, these monofloral honeys might exhibit effects on human health and act positively as prebiotics.

## 1. Introduction

Honey, a food produced by bees (*Apis mellifera*), represented for millennia the only available concentrated sugary food and in some civilizations, such as that of the ancient Egyptians, jars of honey were placed next to mummies, while the ancient Greeks considered it “food of the Gods”. Honey-based recipes were also developed for medical use, and for the production of ointments for the treatment of sores and wounds [1]. With the discovery of sugar cane and sugar beet, honey was gradually supplanted and only recently, by virtue of its recognized therapeutic properties, is it making a comeback. International standards specify that “honey may be designed according to floral or plant source if it comes wholly or mainly from that particular source and has the organoleptic, physicochemical and microscopic properties corresponding with that origin” [2]. In Italy, the legal definition of honey is included in Article 1 of the Legislative Decree 179/2004 and production is about 23,300 tons/year [3]. Honey represents a complete food irrespective of the age. Its characteristics might be affected more by their botanical origin than by their geographic provenance, climate, soil acidity, or other environmental conditions. It is a very complex product, which composition includes more than 200 constituents, such as enzymes, proteins, polyphenols, minerals, free amino acids, vitamins, fructose, and glucose, these last representing the main substances contributing to its high sweetening power [4]. Phenolic acids (1.5–4.2%) and flavonoids (1.2–2.5%) represent polyphenols [5] who serve as powerful antioxidants, provide the honey health beneficial effects and affect its color, taste and aroma [6]. The floral specificity is one of the parameters affecting its therapeutic properties.

The antidiabetic property of honey, although known, is not so widely studied, compared to the numerous types of monofloral honeys existing. Diabetes mellitus (DM) is a chronic metabolic disorder who is fast becoming epidemic in some world countries also due to increase in ageing population and to the suffering of the healthcare providers, especially in poorly developed countries [7]. DM individuals also are subjected to higher risks for microvascular complications, heart attack, and stroke than normal. Representing DM one of the major causes of illness and mortality; nowadays a renewed interest let to investigate the health benefits of herbs and natural products—including honey—in the management of DM. As reported by Nasrolahi et al. (2013) [8] antidiabetic drugs in combination with honey could improve glycemic control, enhance antioxidant defenses, and decrease oxidative damage thereby leading to a reversion of the beta-cell degeneration in pancreas and also enhancing the insulin production as well as reducing the insulin resistance towards the glucose moieties in circulation. Monofloral honeys, such as citrus and thyme ones, showed to act beneficially on the glycemic index (GI); on the other hand, the serum insulin levels were significantly lower after the consumption of the chestnut honey [9]. Studies on rats demonstrated that the honey of *Moringa oleifera* [10] might control the level of the GI. Gourdomichali and Papakonstantinou (2018) demonstrated that fir and chestnut honeys gave medium GI values (59 and 66, respectively, on glucose scale), while citrus, heather, pine and thyme honeys provided high GI (>70 on glucose scale) [11]. *Fabaceae* honeys inhibited the glucosidase activity [12] due also to the presence of polyphenols, which generally can also decrease the starch digestibility [13] and reduce the level of glucose [14].

Among its multiple functional properties, the effect of the honey against the growth and surface attachment ability of pathogens, prodromal to the formation of biofilms is not so widely studied. This last aspect is of noticeable importance from a health point of view. Biofilm formation is a self-protective mechanisms exhibited by bacteria that aggregate to create a complex structure to resist to severe environmental conditions. This gives rise to an increase of their surface attachment ability and a higher population density, with the production of extracellular polymeric substances (EPS) and a chain of chemical, physical, and metabolic processes that lead also to an increase of pathogenic aspects. [15], including its resistance to the conventional antimicrobial agents and to phagocytosis. In such way, they become more difficult to eradicate the biofilm from living hosts [16]. Honey was effective in inhibiting the formation of biofilms of *Pseudomonas aeruginosa* and *Klebsiella pneumoniae* [17], oral streptococci [18], *Proteus mirabilis*, and *Enterobacter cloacae* [19]. Stojkosska et al. (2019) produced alginate hydrogels with silver nanoparticles (AgNPs) and honey components that acted against multidrug-resistant bacterial strains causing nosocomial wound infections [20]. Among monofloral types, manuka honey is one of the most studied. It can inhibit the biofilm formation of bacteria [21]. Tualang honey demonstrated antibacterial activity against *Acinetobacter baumannii*, and could potentially be useful as an alternative therapeutic agent against such microorganisms [22].

Scientific community focused the attention also on the positive effect of honey on probiotics. Chestnut honey could increase the growth of *Lactobacillus acidophilus* and *Lacticaseibacillus rhamnosus*, but its effects could affect also some probiotics properties [23]. Similarly, lime honey enhanced the probiotic properties of *Lactobacillus acidophilus* and *Lacticaseibacillus rhamnosus*, including auto-aggregation and surface hydrophobicity, and might have both direct effects on human health, and indirect benefits mediated by beneficial microorganisms [24].

Therefore, the aim of our work was to evaluate the content of total polyphenols in some Italian monofloral honeys, as well as the capacity exhibited by these honeys to inhibit the α-glucosidase activity, one of the proof ascertaining the in vitro antidiabetic properties of a product. In addition, the effects of the honeys on pathogenic bacteria was assessed. In particular, we evaluated the influence of the honeys on the biofilm formation, on the bacterial adhesion and their effect on mature biofilms. Concurrently, we considered the metabolic changes occurring due to the presence of the honeys with respect to untreated cells. Finally, we evaluated if the presence of the honeys in the culture medium could affect the growth of different probiotic strains.

## 2. Materials and Methods

Different types of commercial organic monofloral honey: fir (*Abies* spp., origin: Tuscany, Veneto, Trentino, Friuli, Italy), strawberry tree (*Arbutus unedo* L., origin: Tyrrenian regions of Italy), ivy (*Hedera helix* L., origin Tuscany, Veneto), tree of heaven (*Ailanthus altissima* Mill., Swingle, origin: Tuscany, Veneto), sulla (*Sulla coronaria* (L.) Medik., origin: Sardinia and Southern-Central Italy), cardoon (*Cynara cardunculus* L., origin: Sardinia), rhododendron (*Rhododendron* spp., origin: Northern Italy) were purchased by an Italian company (Thun, Trento, Italy). The company provided to make the opportune analyses before placing them on the market. Honey samples were stored at 4 °C in the dark until analyzed. They did not show any crystallization, thus were perfectly suspended by mixer in deionized water and phosphate buffer solution (1 g of honey dissolved in 4 mL of solution) and filtered (0.45 μm, Millipore, Merck Life Science, Milano, Italy) before the biochemical analysis and the microbial tests, respectively.

### 2.1. Biochemical Analysis

#### 2.1.1. Determination of Total Polyphenols Content

Total polyphenols content was evaluated using the Folin–Ciocalteu phenol reagent [25]. The absorbance at λ = 760 nm was determined at room temperature through a Cary UV/Vis spectrophotometer (Varian, Palo Alto, CA, USA). Gallic acid represented the standard. Results were expressed as μg of gallic acid equivalents (GAE)/g of the product ± standard deviation (SD). The concentration range for standard curve was between 34.02 μg and 680.04 μg.

#### 2.1.2. α-Glycosidase Inhibition Assay

The test was performed using the methods of Sharp et al. [26] and Watson et al. [27]. Briefly, a 5 mg/mL solution of α-glycosidase (Sigma-G5003, from *Saccharomyces cerevisiae*, 100 U/mg of protein, Milano, Italy) and 1 mM *p*-nitrophenyl-α-D-glucopyranoside (Sigma, Milano, Italy) solution were prepared in 20 mM phosphate buffer (pH 6.0). The reaction was carried out at 37 °C using 10 μL enzyme, 25 μL substrate and 10 μL extract for 10 min, in 80 μL total volume. Absorption was measured at 400 nm after the addition of 80 μL 0.1 M Na_2_CO_3_. A control reaction was performed using 10 μL of aqueous 1% dimethyl sulfoxide (DMSO) of acarbose. The assay was performed in triplicate, and percent inhibition was plotted against concentration to calculate the concentration need to inhibit at 50% the activity of the α-glycosidase (IC_50_).

### 2.2. Antibacterial Properties of the Honeys

#### 2.2.1. Microorganisms and Culture Conditions

*Acinetobacter baumannii* ATCC 19606, *Escherichia coli* DSM 8579*, Listeria monocytogenes* ATCC 7644, *Pseudomonas aeruginosa* DSM 50071, and *Staphylococcus aureus* subsp. *aureus* Rosebach ATCC 25923 were used as test bacterial strains. Bacteria were cultured in Luria Broth for 18 h at 37 °C and 80 rpm (Corning LSE, Pisa, Italy) before the microbial analysis. *A. baumannii* was grown at 35 °C at the same conditions.

#### 2.2.2. Minimal Inhibitory Concentration (MIC)

The MIC of each honey was evaluated by the resazurin microtiter-plate assay [28]. Multiwell plates were prepared in triplicate and incubated at 37 °C for 24 h. *A. baumannii* was grown at 35 °C at the same conditions. The lowest concentration at which a color change occurred (from dark purple to colorless) revealed the MIC value.

#### 2.2.3. Biofilm Inhibitory Action of the Honeys

The capacity of the honeys to affect the biofilm formation by the pathogenic bacteria was evaluated in flat-bottomed 96-well microtiter plates [29]. The overnight bacterial cultures were adjusted to 0.5 McFarland (1.5 × 10^7^ cells/mL. Densitometer cell density turbidity 0.3–15.0 McFarland, CAMLAB, Cambridge, United Kingdom) with fresh culture broth before the test, 10 μL of the diluted cultures were distributed in each well, 5.71 μL/mL and 11.42 μL/mL of each honey- and Luria-Bertani broth were added, to have a final volume of 250 μL/well. Microplates were entirely enclosed with parafilm tape, to avoid the evaporation of material included in the wells, and incubated for 48 h at 37 °C (except *A. baumannii* that was incubated at 35 °C). Planktonic cells were removed, and the attached cells were gently washed twice with sterile PBS. Two hundred μL of methanol was added to each well and retained for 15 min to fix the sessile cells. Methanol was discarded, and each plate was left to dry the samples. The staining of the adhered cells was performed with 200 μL of 2% *w/v* crystal violet solution added to each well and discarded after 20 min. Wells were lightly washed with sterile PBS and left to dry. Two hundred μL of glacial acetic acid 20% *w/v* were added to let the release of the bound dye. The absorbance was measured at λ = 540 nm (Cary, Varian, Milano, Italy). The percent value of adhesion was calculated respect to control (cells grown without the presence of the samples, for whose we assumed an inhibition rate = 0%). Triplicate tests were done, and the average results were taken for reproducibility.

#### 2.2.4. Effect of the Honeys on the Bacterial Adhesion Ability

The capacity of the honeys to affect the bacterial adhesion was evaluated in flat-bottomed 96-well microtiter plates, modifying the method described by Caputo et al. (2020) [29], with the addition of the two concentrations of honey, 5.71 μL/mL and 11.42 μL/mL, after 2 h of bacterial growth. The growth continued until 48 h. The successive steps of the experiment, including the calculation of the percent value of adhesion were performed as indicated in Section 2.2.3.

#### 2.2.5. Action of the Honeys on Mature Bacterial Biofilm

The capacity of the honeys to affect the mature biofilm, considered after 24 h of bacterial growth was evaluated in flat-bottomed 96-well microtiter plates following, the same protocol described in Section 2.2.3 [29]. After 24 h of bacterial growth, planktonic cells were removed, and the two concentrations of the honeys, 5.71 μL/mL and 11.42 μL/mL of each sample and Luria-Bertani broth were added, to have a final volume of 250 μL/well. After further 24 h of incubation, the sequential steps of the experiment, including the calculation of the percent value of inhibition with respect to the untreated bacteria were performed as indicated in Section 2.2.3.

#### 2.2.6. Metabolic Activity of Biofilm Cells

The effect of two concentrations, 5.71 μL/mL and 11.42 μL/mL of the honeys (prepared as above described)—added at the beginning of the bacterial growth, after two hours of incubation and after 24 h of incubation—was also evaluated on the metabolic activity of the bacterial cells through the 3-(4,5-dimethylthiazol-2-yl)-2,5-diphenyltetrazolium bromide (MTT) colorimetric method [29,30], using 96-well microtiter plates. The overnight bacterial cultures were adjusted to 0.5 McFarland and microtiter plates were prepared as described in the Section 2.2.3. After 48 h total of incubation, bacterial suspension, representing the planktonic cells, was removed and 150 μL of PBS and 30 μL of 0.3% of MTT (Sigma, Milano, Italy) were added, keeping microplates at 37 °C (except than *A. baumannii* that was incubated at 35 °C). After 2 h, the MTT solution was removed and two washing steps were performed with 200 μL of sterile physiological solution. Then, 200 μL of dimethyl sulfoxide (DMSO) were added to let the dissolution of the formazan crystals that were measured at OD = 570 nm (Cary, Varian) after 2 h. Triplicate tests were carried out and the average results were taken for reproducibility.

### 2.3. Effect of the Honeys of the Growth of Probiotics

*Lactobacillus acidophilus*, *Lactobacillus gasseri*, *Lacticaseibacillus casei*, *Lactiplantibacillus plantarum*, and *Lacticaseibacillus rhamnosus*, bought in a local apothecary, were grown in De Man, Rogosa and Sharpe (MRS) medium (Sigma-Aldrich, Milano, Italy) for 18 h at different temperatures depending on the strain: in particular, *L. acidophilus*, *L. gasseri*, and *L. rhamnosus* were grown at 37 °C; *L. casei* and *L. plantarum* were grown at 30 °C. All strains were used as inoculum. The eight honeys, used as carbon source, were dissolved in sterile MRS broth without glucose (Liofilchem srl, Roseto degli Abruzzi, Italy) to have two final concentrations corresponding to 1% and 2% (*w/v*). The influence of the presence of the honeys was assessed respect to the control, grown in the presence of glucose as control carbon source (MRS only). After 24 h of incubation in flat-bottomed 96-well microtiter plates, the growth was evaluated at OD_600_ nm (Cary, Varian). Triplicate tests were carried out and the average results were taken for reproducibility.

### 2.4. Statistical Analysis

The MATLAB suite was used for the calculations. Data were expressed as mean ± standard deviation of triplicate measurements. The analysis correlated the normalized values of inhibitory activity exhibited by the honeys on microbial biofilm (using the data giving rise from the Cristal Violet test) and the normalized inhibitory activity of the honeys on the microbial metabolism (using the data giving rise from the MTT test).

## 3. Results and Discussion

### 3.1. Total Polyphenol Content

The content of total polyphenols (TPs) present in the various Italian monofloral honeys analyzed is shown in Table 1. It was very variable, ranging from 110.46 μg/g (in rhododendron honey) up to a value of 552.29 μg/g (in strawberry tree honey). Such data were lower than those found for various plurifloral honeys, observed by Vela et al. [31], but we should obviously take into account the nature of the honey (multifloral or monofloral), of the plant of origin, the geographical area and the pedo-climatic conditions, which also can influence some biochemical characteristics of the honey. Petretto et al. [32] analyzed some biochemical and physico-chemical characteristics of some Sardinian monofloral honeys and observed data similar to what we found for strawberry tree honey and higher for cardoon honey. Perna et al. [33] observed that the sulla honey produced in Southern Italy exhibited a total polyphenol content lower than that contained in the honey analyzed in our tests. Ivy honey exhibited a higher total polyphenol content than that present in the honey analyzed by Kavanagh et al. [34]. Our sulla honey contained a quantity of total polyphenols higher than that found by Perna et al. and Pirichero et al. [33,35], the latter also observed a lower TP content also in the honey from tree of heaven compared to our sample (93.72 vs. 220.62 μg/g, respectively). The honey of rhododendron showed a TPs content of 110.46 μg/g. Such value fits perfectly with the range of values found by Silici et al. [36] who, analyzing numerous honeys of rhododendron from different areas of Turkey, observed that the amount of TPs varied from 2.98 to 1113.3 μg/g, to testimony that the biochemical characteristics of a specific monofloral honey can vary not only in relation to the year but even to the region within the same nation, as also demonstrated by Gul and Pehlwan [37] who found—in the strawberry tree and rhododendron honeys produced in Turkey—a total polyphenol content higher than that observed in our research.

The analysis of polyphenols can represent a very promising way to study the floral and geographical origins of honeys not only to add important dowels to their quality characteristics but also to identify those with higher healthy properties, including the inhibitory effect on pathogenic bacteria [38].

### 3.2. α-Glycosidase Inhibitory Activity

Therapies against type-2 diabetes involve the use of drugs as enzyme inhibitors in order to decrease glucose adsorption in the gut. However, certain foods, including honey, can inhibit this enzyme, representing a natural source of inhibitors [39,40]. The inhibition of enzymes like α-glycosidase, involved in the carbohydrate digestion, might represent a noteworthy method for decreasing postprandial hyperglycemia. In our experiments, all types of monofloral honeys resulted capable to inhibit the action of α-glycosidase. Results are shown in Table 1. By the whole, the amount of honey necessary to inhibit at 50% (IC_50_) the action of α-glycosidase was never superior to 34.07 mg/mL and in five honeys (tree of heaven, sulla, fir and rhododendron) the values of IC_50_ was less than 30 mg/mL. The honey of sulla even demonstrated the best capacity to inhibit the α-glycosidase activity, with an IC_50_ value of 20.2 mg/mL. We did not find any correlation between the content of total polyphenols and the α-glycosidase inhibition. The honey of strawberry tree, which exhibited the highest content of polyphenols, showed a weak capacity to affect the α-glycosidase activity, with an IC_50_ value of 32.7 mg/mL. Therefore, the honey of sulla, with the lowest content of polyphenols (182.4 μg/g of the product) exhibited the best capacity to inhibit the α-glycosidase; however, the honey of cardoon, which also had a polyphenol content practically the same of that of sulla, was the weakest in inhibiting the action of α-glycosidase, with an IC_50_ value = 34.5 mg/mL.

Probably, in our case, such capability could not be merely related to the amount of total polyphenols, but also to the presence of other important molecules, conversely to what indicated by Zaidi et al. (2019) who, analyzing different types of monofloral honeys, observed a good correlation between total polyphenol content, anti-inflammatory activity and α-glycosidase activity [12], although none of their samples were similar to our samples. Moreover, Ali et al. (2020) observed a correlation between the content of polyphenols and the α-glycosidase activity in the honeys, although a stingless bee produced them [41]. This could support also the thesis that the quality of a honey, and therefore also its biochemical characteristics and biological properties, can be related to the botanical origin and type of bee producing the honey [41,42]. Krishnasree and Ukkuru [43] evaluated the antidiabetic capacity of the honey obtained from five bee species, measuring their glycemic index, the glycemic load and in vitro antidiabetic activity, such as the α-glucosidase inhibition assay. They reported that the honey produced by *A. mellifera* caused an inhibition of 79.86% and 69.17% in raw and processed honeys, respectively. Therefore, in each case, all monofloral honeys aroused considerable interest, as they are susceptible, when included in a balanced diet, to act as antidiabetic factors.

### 3.3. Antibacterial Activity

The minimal inhibitory concentration (MIC) of the honeys, needed to block the growth of the five species of bacteria, was evaluated by using the resazurin test. Results are reported in Table 2.

The antibacterial effects of honey are thus a complex action of several factors that are present in the honey [44,45] and depend for instance on the bees’ source of nectar, the location of the flowers and related weather conditions [46,47]. Often, honey has exhibited an antibacterial effect on Gram-negative bacteria, and more pronounced against Gram-positive bacteria [48,49]. Honey can act against bacteria through its capacity of generating hydrogen peroxide but also through its very complex composition, which has more than 180 components [50]. In our experiments, the antibacterial activity of monofloral honeys was tested against two Gram-positive bacteria, *L. monocytogenes* and *S. aureus* and the Gram-negative bacteria *P. aeruginosa*, *E. coli*, and *A. baumannii*. The tests carried out concerned the determination of the MIC (see Table 2) and, subsequently, the analysis of the effect that the different monofloral honeys could exert on the ability of bacteria to act on biofilm and metabolism of the bacterial cells present within biofilm (Table 3, Table 4 and Table 5). The ability to block bacterial growth exerted by the honeys was not associated with the different cell structure, although Gram-positive and Gram-negative bacteria express a different resistance/sensitivity to antibiotics, also according the different cell wall structure [51]. We verified that the MIC ranged between 20 to up 50 μL/mL. As already observed with regard to the inhibitory action exerted by honeys on α-glycosidase, the antimicrobial efficacy did not seem to be linked to the total polyphenol content, or at least not always related to such parameter, but could be linked also to the presence of hydrogen peroxide, glucose oxidase and catalase, notoriously present in the product [38]. Strawberry tree honey, which had the highest content of TPs, did not exhibit a correlatable inhibitory activity against the microorganisms tested, especially *P. aeruginosa* (MIC = 40 μL/ mL) and was more effective vs. *E. coli* (MIC = 25 μL/mL). In contrast, rhododendron honey, which had the lowest TPs content (110.46 μg/g of product), although exhibiting less inhibitory strenght against *S. aureus*, was more powerful against *E. coli* (MIC = 25 μL/mL) and mainly *A. baumannii* (MIC = 20 μL/mL). This was in contrast with Stagos et al. (2018) who found a correlation between the amount of TPs and some biological properties of the honey they analyzed, including the antibacterial effect [52]. The activity of the rodhodendrum honey agrees with the results of Silici et al. [36], which confirmed the powerful antimicrobial effect of this honey against *P. aeruginosa*. The antibacterial activity of fir honey against *S. aureus* and *P. aeruginosa* was higher than that reported by Melliou and Chinou [53] and Broznic et al. [54].

### 3.4. Activity of Honeys on Biofilm

#### 3.4.1. Activity of Honeys on Biofilm Formation and Bacterial Metabolism

The capacity of the monofloral honeys to affect the biofilm (formation of biofilm, adhesion of bacteria to the wells, mature biofilm) and the metabolism of microbial cells was quantified by colorimetric analysis with crystal violet and MTT, respectively. Results are shown in Table 3. The test was carried out using two concentrations of each sample, 5.71 μL/mL and 11.42 μL/mL, both below that needed to inhibit the microbial growth.

Overall, at the higher concentration used in the tests, the honeys proved to inhibit the bacterial capacity to form biofilms, with inhibition percentages that in several cases higher than 90%.

*L. monocytogenes* was generally the most sensitive strain to the inhibitory action of all the honeys, with percentages that in some cases reached (in the presence of ivy honey) and even exceeded 90% (in the presence of fir and cardoon honeys). *P. aeruginosa* showed almost the same weakness towards the honeys tested, which determined an inhibitory effect on the biofilm formation that reached percentages of up to 93.41%. However, the presence of tree heaven honey at the lower concentrations tested was unable to determine any effect on its biofilm but, at higher concentration, these honeys demonstrated a certain inhibitory capacity, which reached 49.14%. Higher weakness was exhibited by sulla (34.99%) and especially by rhododendron honey, which inhibitory effect did not exceed 14.60%. *A. baumannii* also was very sensitive to the action of the honeys and, except in the case of strawberry tree, all the other honeys determined an inhibitory action that never went below 26.38% (in the case of tree of heaven) and reached 83.46%. *E. coli* was the only bacterial strain showing albeit slightly greater resistance to the action of honeys; however, when it was sensitive to the honeys (except rhododendron) when we used the highest concentration, and percentages of inhibition up to 72.92% (in the case of sulla honey) were observed. When added at zero time, some honeys were more effective than others in inhibiting the formation of biofilm *ab origine*, regardless of the type of bacteria used as a tester. In fact, fir honey determined an inhibitory effect ranging between 44% (5.71 μL/mL vs. *E. coli*) and 92.03% (11.42 μL/mL vs. *L. monocytogenes*); cardoon honey, caused an inhibition almost always higher than 80%, reaching values, even of 93.41%, except when tested against *E. coli* (inhibition: 15.41%). The ivy honey inhibited the formation of biofilm at percentages between 54.71% and 89.93% when tested at the higher concentration. Furthermore, it should be underlined that the honeys did not exert an inhibitory effect depending on the bacterium (Gram-negative or Gram-positive). Thus, the Gram-negative bacteria *A. baumannii*, *P. aeruginosa*, and *E. coli,* as well as the Gram-positive *L. monocytogenes* and *S. aureus,* treated with strawberry tree honey showed a different reaction. Therefore, sulla honey affected in a similar way the formation of the biofilm by *E. coli* and *L. monocytogenes* (72.92% and 72.29, respectively) as well as by *A. baumannii* and *P. aeruginosa* (32.12% and 34.99% of inhibition, respectively).

The analysis of correlation among the normalized values of inhibitory activity on microbial biofilm and those of the normalized inhibitory activity on the microbial metabolism (Corr-coeff = −0.23), evidenced that often the honeys capable to inhibit the formation of biofilm did not act in the same way on cellular metabolism, meaning that it may work differently than how it behaves on biofilm. Fir honey represented the most striking example. In fact, it, in the face of a truly remarkable efficacy in blocking the formation of the biofilm of *L. monocytogenes* (91.54% of inhibition), did not equally act on its cellular metabolism (giving in this case a percentage of metabolic inhibition equal to zero). Ivy honey exhibited the same behavior. Other honeys, on the other hand, proved to act as good inhibiting agents both on biofilm formation and on bacterial metabolism: cardoon honey resulted capable to inhibit almost completely the formation of the biofilm by *P. aeruginosa* (93.41% inhibition) but also to block at 81.53% the metabolism of the bacterial cells within the biofilm. On the contrary, its action was very effective in inhibiting the biofilm formation of *L. monocytogenes* (93.27%) but not in inhibiting its metabolism. In the case of *E. coli*, we observed a diverse situation: a weak inhibitory action on the formation of the biofilm (15.41%) was offset by a much more effective inhibitory action (71.49%) on the metabolism of the cells present within the biofilm.

Our results confirmed the capacity of monofloral honeys to inhibit the formation of biofilm, which can be due to the presence of different components capable to act on structural aspects of the bacteria and/or on its metabolism [55]. We proved a different inhibitory activity of the honeys vs. *S. aureus* [55]; however, strawberry tree honey did not act against *S. aureus* and *P. aeruginosa* conversely to that reported by da Silva et al. [56]. In a certain way, the MTT test confirmed such aspect, indicating that, when the honey did not reach to inhibit the formation of biofilm, it could act on its metabolism. The capacity of the honey to inhibit the biofilm formation depends on the microorganisms, further than the type of honey. Thus, some types of honey, such as the manuka one, not only do not induce a significant cellular lysis of the methicillin-resistant *S. aureus,* such as that we used in our experiments, but also causes few surface changes [57]. On the contrary, when manuka honey act against *P. aeruginosa*, this could cause widespread structural damage and large membrane bubbles, leading to cell lysis and bacterial death [57]. These mechanisms are not exclusively attributable to the manuka honey, and several honey varieties produced morphological and structural alterations on bacteria as one of their first effects [58].

#### 3.4.2. Inhibitory Action of Honeys on the In Vitro Bacterial Adhesion and Bacterial Metabolism

The capacity of adhesiveness and biofilm formation by all microorganisms are well-orchestrated processes that answer to a wide range of cellular and environmental signals [59]. Our aim was also to evaluate the effect of monofloral honeys also on the adhesive property of pathogenic strains, by adding the honeys after 2 h from the incubation. Results are shown in Table 4. The behavior exhibited by the honeys and the sensitivity of the bacteria, were different. Some honeys, such as tree of heaven honey, managed to inhibit the adhesion process in vitro, with percentages, which reached 79.46% and 84.27% (vs. *S. aureus* with 5.71 and 11.42 μL/mL, respectively).

Compared with their effectiveness to inhibit the biofilm formation, not always the honeys were unable to exhibit the same inhibitory strength on the adhesive capacity of bacteria. Sulla honey was still capable to exert an inhibitory effect when added after two hours of incubation, although with less efficacy. On the contrary, tree of heaven honey acted much more on the adhesion process: *S. aureus* that demonstrated a good resistance to the action of this honey (with an inhibition of its biofilm not exceeding 26.13%), exhibited greater sensitivity when this honey was added after two hours (inhibition: 76.64%). Moreover, if 5.71 μL/mL of this honey did not affect the ability of *P. aeruginosa* to form the biofilm, the same concentration was able to inhibit by 34.81% the ability of this microorganism to adhere to the multiwell plates. The inhibitory effectiveness of the honey of tree of heaven against *E. coli* practically doubled, passing from 38.92% to 73.66%. Rhododendron honey did not act against the biofilm formation by *E. coli*; therefore, it was capable to inhibit by 66.63% its adhesion. The inhibitory efficacy of the strawberry tree honey against *S. aureus* doubled (20.80 and 43.81% of inhibition, respectively). Sulla honey maintained its efficacy or even increased such inhibitory capacity (for instance in the case of *A. baumannii*, against which its inhibitory capacity increased from 32.12% to 51.77%). The capacity of *A. baumannii* to grow as biofilm on abiotic surfaces plays an important role in causing nosocomial infections [60,61]. Once again, the action of the honeys was different on the bacterial metabolism compared to their capacity to act on the adhesion process. However, the correlation between the inhibitory action on the bacterial adhesion and the bacterial metabolism resulted higher (Corr-coeff = 0.41). In some cases, the effect of the honey on the microbial metabolism did not correspond to its effectiveness against the adhesion process: the honey of tree of heaven, for instance, was more effective in inhibiting the adhesion of *E. coli* and *L. monocytogenes* (73.66% and 69.49%, respectively) than the metabolism of their cells (25.52% and 46.78%, respectively). On the contrary, the honey of cardoon, capable to inhibit the adhesion of *A. baumannii* by 19.95%, acted with much more efficacy on its metabolism (44.97%).

#### 3.4.3. Action of Honey on Mature Biofilm

As described in the Section 2, we provided to evaluate the potential effect of the honeys on mature biofilms, by adding the honeys after 24 h of bacterial growth, when the biofilm has been already formed by the microorganisms and the metabolism of the bacterial cells is different respect to the initial state. Results are shown in Table 5.

By the whole, the honeys were capable to act also on a mature biofilm, maintaining, in some cases, a strong efficacy of inhibitory action on the metabolism, as evidenced by the analysis of correlation (Corr-coeff = 0.66).

The tree of heaven honey exhibited an inhibitory strength against *S. aureus* similar to that shown in inhibiting its adhesion (76.64% and 84.27%, respectively). Honey of ivy even increased its action against such bacteria, so that the inhibition exhibited by this honey passed from 51.76% to 64.96%. Other honeys, on the other hand, exhibit a greater efficacy of inhibitory action if placed in contact from the beginning of their growth with pathogenic microorganisms. In our tests, we often observed that although an action on the mature biofilm did not correspond to a similar effectiveness on the metabolism of the cells present within the mature biofilm, in any case the honeys tried to counteract the bacterium (making the comparison with the results of the test with crystal violet). There have been few negative exceptions in this regard. All honeys, for example, proved to be completely ineffective against *E. coli*, both as regards the action on the mature biofilm and as regards a possible inhibitory influence on bacterial cellular metabolism. Some of the honeys that were effective on the mature biofilm produced by *A. baumannii* exhibited a much less incisive action—in some cases completely ineffective—in counteracting the metabolic changes within the bacterial cells present within the biofilm. On the contrary, in the case of *L. monocytogenes*, we observed that the honeys maintained a good efficacy of action on the mature biofilm, although they did not always do the same on the metabolism of this bacterium. Evidently, we could hypothesize that honeys, like several substances of natural origin, have specific action, so they can act on microbial steps not necessarily linked to cell metabolism (for example by working on the membrane of the bacterium but not on its enzymes). In some cases, honey could have a broader and more diversified spectrum of action, and therefore can act both on the structure of microbial cells and biofilms and on the metabolism of the cells present within the biofilm. Ivy honey provoked 50.98% of inhibition on the mature biofilm formed by *L. monocytogenes* and 53.80% on its cellular metabolism; similarly, the presence of sulla honey determined a percentage of inhibition practically equal against *L. monocytogenes* both in the crystal violet test (37.47%) and in the test conducted with MTT (36.45%). The action of almost all honeys was effective against the mature biofilm and metabolism of *P. aeruginosa* and only in two cases (in the presence of rhododendron and sulla honeys, but at the lower concentration), the efficacy of action of the honey did not correspond to an efficacy of action on microbial metabolism. This result could be important indeed. We know, in fact, that *P. aeruginosa* is a very virulent wound pathogen and is commonly isolated from the poly-microbial biofilms found in chronic wounds [62,63]. Infections caused by *P. aeruginosa* are particularly difficult to cure owing the intrinsic mechanisms of antibiotic resistance of the organism, as well as the structure of its biofilm matrix that impedes the penetration of the biofilm, and so the associated chronic wound infections often do not respond to treatments with conventional antibiotics [64].

The anti-biofilm activity in vitro has been reported for some honeys, such as the honey of manuka who could avoid the formation of biofilms produced by different pathogens, including *S. aureus* and *P. aeruginosa*, although with different levels of action [17,57,64,65]. This confirmed, once again that—with some exceptions—where honey could not prevent the formation of the biofilm, it influenced the metabolism of the bacterial cells present within the biofilm (initial, mature, or on its adhesion), which, through processes of metabolic changes, led them to become much more resistant to the action of antibiotics and therefore related infections are more difficult to eradicate.

Many of the honeys we tested were capable to act at different level on this strain, so that we observed still inhibition up to 64.96% (given by the ivy honey in the text with cristal violet) and 43.89% inhibition on its metabolism caused by the presence of the same honey. Several studies have ascertained the antibacterial properties of honey. However, the lack of widespread data illuminating on the mechanisms through which honey interferes with bacteria somewhat limits its application as antibacterial agents [17]. As a very complex substance, the honey could cause specific and distinct effects on microorganisms. However, as our data indicated, some cellular targets might be broadly not specific. The overall analysis of the behavior exhibited by honeys in the three events taken into consideration (inhibition on the formation of the biofilm, adhesion of bacteria to the walls of the microplates, action of the honeys on mature biofilms, and related metabolism) permits some useful considerations for possible uses of these honeys in contrast to these pathogenic microorganisms. Some honeys, such as fir honey, were usually effective, so much so that they not only maintained an effective action on the mature biofilm but also were still able to inhibit cellular metabolism, even with a greater efficacy of action on cellular metabolism. This was the case of *A. baumannii* where the percentage of inhibition passed from 9.52% to 40.43% even up to 85.19% in the test done with MTT on the 24-h biofilm. Therefore, the behavior exhibited by the honeys was not always linear but somewhat fluctuating. Thus, in the test carried out with crystal violet the effectiveness of the honey of sulla was greater in countering the adhesion of *A baumannii* while, in the test with MTT, the same honey was able to inhibit the metabolism more effectively the microbial metabolism if added at the beginning of growth (66.30% inhibition) or after 24 h of growth (31.28% inhibition) rather than if added after two hours of growth, when its efficacy on the cellular metabolism resulted only at 13.48%. Rhododendron honey, which also decreased its efficacy against *A. baumannii* (with inhibition percentages that went from 69.68% to 51.67% up to 11.43% on mature biofilm, in the violet crystal test), instead exhibited an oscillating trend as regards its action on cellular metabolism. In fact, the results of MTT test demonstrated its effectiveness on the metabolic processes occurring for the formation of the biofilm and especially on the mature biofilm, while it had little inhibitory effect on the metabolic pathway allowing the adhesion of *A. baumannii.* In the case of *L. monocytogenes,* the action of the honeys on the formation of the biofilm and on the adhesion process was more effective than that exhibited on the mature biofilm, but in any case all the honeys retained a certain efficacy also on the mature biofilm, even if there was not always a similar correspondence of behavior on the microbial cellular metabolism, on which, in some—fortunately few—cases the honeys (thistle and strawberry tree) were ineffective even at the highest concentration.

By the analyses of correlation, it could be said overall that the action of honeys became more effective both on the biofilm and on the metabolism of the cells contained within the biofilm concurrently to the time. Thus, we observed a negative value (Corr-coeff = −0.23) when the honey was added at the beginning of the bacterial growth. The correlation coefficient rose to settle on a positive value (Corr-coeff = 0.41) when the honey was added to the culture when the bacterial growth had already begun but not such as to have determined the definitive formation of the biofilm. The most important aspect, in our opinion, is represented by the fact that the honeys, albeit obviously in a different way, managed to exert a certain inhibitory influence both on the biofilm and on the bacterial metabolism in the mature biofilm phase (Corr-coeff = 0.66), a more difficult situation to fight.

The action exhibited by the honeys against the biofilm formation and metabolism of some pathogens—in particular *S. aureus* who are associated also to the insurgence of diseases such as the Alzheimer disease—stimulates us to carry further investigation about the antimicrobial effect of the honeys against other pathogens involved in neurological pathologies, first *Porphyromonas gingivalis*. This will allow underlining accent about the healthy role of the honey in the diet style, in particular that of the elderly people, more affected by metabolic pathologies and more sensitive to some diseases affecting the nervous system [66,67,68].

### 3.5. Effect of Monofloral Honeys on Probiotics Growth

**Figure 1:**
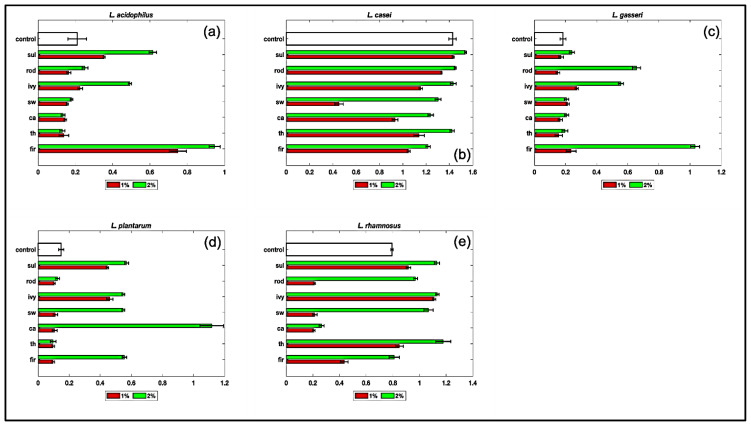
Effect of the monofloral honeys—added to a free glucose MRS at 1% and 2%—on the growth of five *Lactobacilli: L. acidophilus* (subfigure **a**); *L. casei* (subfigure **b***)*; *L. gasseri* (subfigure **c**); *L. plantarum* (subfigure **d**); *L.rhamnosus* (subfigure **e**). Results are expressed as OD 600. Each control was grown in the conventional MRS. Legend: ca: cardoon honey; fir: fir honey; ivy: ivy honey; rod: rhododendron honey; sul: sulla honey; sw: strawberry tree honey; th: tree of heaven honey. Red bars represent the experiment performed with 1% honey; green bars represent the experiments performed with 2% honey. Experiments were performed in triplicate.

Honey is as a potential prebiotic, since it has oligosaccharides capable to promote the growth of lactobacilli and bifidobacteria, and possess antimicrobial components, which can act synergistically with the probiotics against certain pathogens [69]. Other reported benefits of the honey as prebiotics include heightened probiotic persistence in the GI tract, improved amounts of short chain fatty acids, and augmented resistance to pathogens [70,71]. The honeys were also tested to verify their effect on the growth of microorganisms with proven probiotic properties, *Lactobacillus acidophilus, Lacticaseibacillus casei, Lactiplantibacillus plantarum, Lactobacillus gasseri*, and *Lacticaseibacillus rhamnosus.* The results are shown in the Figure 1a–e.

We used two different concentrations of honeys (final concentration 1% and 2%), resuspended in MRS broth without glucose, and we evaluated if the replacement of glucose with honeys could in some way have some influence, positive or negative, on the growth of probiotics, compared to the control, grown in conventional MRS broth. The behavior looked different according to the microorganism considered and to a lesser extent depending on the honey considered. Ivy, sulla, and mainly fir honey stimulated very clearly the growth of *L. acidophilus* up to four times compared to the control (Figure 1a). Fir honey, in particular, had a very stimulating effect on the growth of the bacterium already at 1%. The growth-stimulating action of monofloral honeys on *L. casei* (Figure 1b) did not seem very incisive, and only the honey of sulla seemed stimulate the growth of that bacterium when added at 2%. Generally, none of the honeys seemed to inhibit the growth of *L. gasseri* respect to the control. Fir, ivy, and rhododendron honey in particular showed to stimulate strongly its growth (Figure 1c). *L. plantarum* was positively influenced during its growth by sulla, ivy, strawberry, and fir. Honey of cardoon stimulated the growth of this bacterium up to six times compared to the control (Figure 1d). The honeys of tree of heaven, ivy, strawberry tree, rhododendron, and sulla emerged as those with the highest growth stimulating effect on *L. rhamnosus* (Figure 1e). The data of our experiments suggested that some honeys, such as fir, ivy, and sulla honey have a broad spectrum of action, since they could stimulate the growth of all the microorganisms tested. Other honeys, such as cardoon honey are more specific. Furthermore, in some cases, the effect of the honeys was usually positive but the increase in terms of growth was not very striking.

At our knowledge, for the first time a study about the potential prebiotic effects (at least their stimulating growth effect) of these honeys all of Italian origin has been carried. Our results are in agreement with Carvalho de Melo et al. (2020) who highlighted the potential prebiotic properties of four monofloral honeys produced by stingless bees in the Brazilian Northeastern semi-arid region on *L. acidophilus* and *Bifidobacterium* [72]. Das et al. (2015) demonstrated a growth-promoting effect of five *Sesamum indicum* honeys on *L. acidophilus* [73] too and Shamala et al. (2000) ascertained a stimulatory effect of a honey produced in a coffee area on the multiplication of *L. acidophilus* and *L. plantarum* indeed [74]. In the future, the evaluation of some specific properties exhibited by the probiotic strains—e.g., the resistance to bile salts, the adhesive in vitro capacity, as well as their antioxidant activity of probiotic bacteria grown in the presence of these honeys—will allow to acquire more information and new applicative scenarios about the positive effect of the honey on human microbiome.

## 4. Conclusions

The monofloral honeys herein studied demonstrated interesting beneficial properties both from a biochemical point of view and about their effect on the pathogens and probiotic microorganisms.

From a biochemical point of view, we have seen that the both polyphenols content and the inhibitory activity on α-glucosidase exhibited by such honeys can constitute interesting features of these honeys. Future steps will be to determine the content of flavonoids and the potential correlation between their content and the functional activity of the honey, both on the α-glucosidase and on the α-amylase activity, a second pillar enzyme involved in the diabetic pathway. The results we obtained from the microbial tests indicated interesting extensive action of these honeys against different pathogens, as well as their capacity to act as stimulating-growth agents toward probiotic strains. It is important to underline the action of the honeys both on the biofilm and on the metabolism of the cells included in the biofilm. The analysis of the correlation coefficients evidenced that such action was as stronger as the age of biofilm, thus the honeys could affect both the adhesion process and mainly the mature biofilm phase, a more complex situation to combat, which not always is exhibited by natural compounds with antimicrobial activity, especially in older people, whose immune defenses are notoriously weaker and more susceptible to microbial attack.

## Figures and Tables

**Table 1:** Total Polyphenols (μg/g) and inhibitory α-glucosidase activity (IC_50_, mg/mL) of the monofloral honeys. Data are mean values ± SD of three determinations.

	Total Polyphenols μg/g (±SD)	α-Glucosidase Inhibition IC50 mg/mL (±SD)
Strawberry tree	552.29 (±22.18)	32.7 (±2.4)
Tree of heaven	220.62 (±8.19)	25.4 (±2.1)
Sulla	182.4 (±11.21)	20.2 (±2.8)
Cardoon	183.95 (±6.3)	34.03 (±3.1)
Ivy	257.07 (±8.73)	1.29 (±4.5)
Fir	386.01(±15.15)	26.8 (±2.3)
Rhododendron	110.46 (±15.21)	28.7 (±2.7)

**Table 2:** Minimal inhibitory concentration (MIC, μL/mL) of the monofloral honeys, needed to block the growth of the five bacterial strains, evaluated by the resazurin test. Data are mean values of three determinations (±SD).

	*A. baumannii*	*E. coli*	*L. monocytogenes*	*P. aeruginosa*	*S. aureus*
Cardoon	30 μL/mL (±3.0)	35 μL/mL (±3.0)	20 μL/mL (±3.0)	25 μL/mL (±5.0)	30 μL/mL (±2.0)
Fir	25 μL/mL (±2.0)	35 μL/mL (±2.0)	20 μL/mL (±2.0)	35 μL/mL (±3.0)	20 μL/mL (±2.0)
Ivy	25 μL/mL (±2.0)	25 μL/mL (±2.0)	25 μL/mL (±2.0)	30 μL/mL (±3.0)	35 μL/mL (±3.0)
Rhododendron	20 μL/mL (±2.0)	25 μL/mL (±2.0)	30 μL/mL (±2.0)	35 μL/mL (±3.0)	>50 μL/mL
Strawberry three	35 μL/mL (±5.0)	25 μL/mL (±5.0)	35 μL/mL (±5.0)	40 μL/mL (±4.0)	35 μL/mL (±3.0)
Sulla	20 μL/mL (±5.0)	20 μL/mL (±5.0)	20 μL/mL (±5.0)	35 μL/mL (±3.0)	45 μL/mL (±3.0)
Tree of Heaven	35 μL/mL (±2.0)	30 μL/mL (±2.0)	25 μL/mL (±2.0)	35 μL/mL (±3.0)	35 μL/mL (±3.0)

**Table 3:** Inhibitory activity of the monofloral honeys, tested at 5.71 μL/mL and 11.42 μL/mL, on the biofilm formation capacity by five pathogenic strains. Results are expressed as percentage (average ±SD) and calculated assuming for the control (untreated bacteria) an inhibition value = zero. Legend: ca: cardoon honey; fir: fir honey; ivy: ivy honey; rhod: rhododendron honey; sul: sulla honey; sw: strawberry tree honey; th: tree of heaven honey. AB: *A. baumannii*; EC: *E. coli*; LM: *L. monocytogenes*; PS: *P. aeruginosa*; SA: *S. aureus.* MTT: data of the inhibitory action exhibited by the honey on bacterial metabolism, evaluated through the MTT test; CV: data of the inhibitory action exhibited by the honeys on the biofilm, evaluated through the Cristal violet assay.

	MTT					CV				
	AB	EC	LM	PS	SA	AB	EC	LM	PS	SA
ca	21.55	59.59	0	80.37	60.66	58.16	0	92.88	93.12	83.86
5.71 μL/mL	(±1.03)	(±2.38)	(±0)	(±0.31)	(±0.95)	(±0.98)	(±0)	(±0.22)	(±0.25)	(±2.71)
ca	26.04	71.49	0	81.53	62.25	82.00	15.41	93.27	93.41	88.33
11.42 μL/mL	(±1.18)	(±1.20)	(±0)	(±0.11)	(±0.30)	(±1.29)	(±1.31)	(±0.19)	(±0.13)	(±0.15)
fir	0	49.74	0	66.10 (±1.35)	42.87	71.42	44.00	91.54	88.00	81.90
5.71 μL/mL	(±0)	(±1.13)	(±0)		(±3.77)	(±3.18)	(±2.05)	(±0.15)	(±0.19)	(±1.46)
fir	9.52	61.85	0	67.67	70.14	76.79	64.96	92.03	89.80	80.07
11.42 μL/mL	(±1.24)	(±0.74)	(±0)	(±0.33)	(±1.00)	(±2.80)	(±4.91)	(±0.23)	(±0.10)	(±0.89)
ivy	7.18	43.17	0	73.06	44.75	65.89	0	87.71	86.26	75.66
5.71 μL/mL	(±1.78)	(±1.42)	(±0)	(±0.56)	(±3.04)	(±1.45)	(±0)	(±0.20)	(±1.02)	(±1.23)
ivy	9.81	48.61	0	71.03	40.12	72.26	54.71	89.93	87.28	78.43
11.42 μL/mL	(±2.43)	(±1.68)	(±0)	(±2.09)	(±0.72)	(±1.06)	(±1.73)	(±0.18)	(±0.37)	(±0.18)
rhod	18.55	8.93	0	52.91	32.65	29.15	0	49.84	0	0
5.71 μL/mL	(±0.57)	(±0.37)	(±0)	(±1.33)	(±1.33)	(±1.90)	(±0)	(±2.19)	(±0)	(±0)
rhod	39.37 (±3.21)	13.98	0	60.44	38.05	69.68	0	65.70	14.60	0
11.42 μL/mL		(±0.78)	(±0)	(±0.56)	(±1.05)	(±2.68)	(±0)	(±1.55)	(±1.95)	(±0)
sul	55.46	23.21	0	57.59	38.13	16.47	41.59	56.79	0	0
5.71 μL/mL	(±0.93)	(±1.93)	(±0)	(±0.43)	(±0.21)	(±2.19)	(±2.08)	(±0.46)	(±0)	(±0)
sul	66.30	44.75	0	62.61	39.03	32.12	72.92	72.29	34.99	1.54
11.42 μL/mL	(±0.85)	(±2.00)	(±0)	(±0.21)	(±0.29)	(±1.71)	(±1.70)	(±0.98)	(±7.79)	(±0.46)
sw	6.23	55.08	0	63.12	22.98	0	0	57.68	9.86	0
5.71 μL/mL	(±2.29)	(±1.26)	(±0)	(±0.27)	(±1.00)	(±0)	(±0)	(±0.60)	(±1.92)	(±0)
sw	16.05	69.72	0	64.32	24.89	72.19	21.51	61.00	10.29	20.80
11.42 μL/mL	(±1.10)	(±1.11)	(±0)	(±0.30)	(±0.23)	(±3.11)	(±1.68)	(±0.69)	(±1.02)	(±2.35)
th	27.07	49.88	0	61.73	27.11	26.38	6.98	65.83	0	1.05
5.71 μL/mL	(±1.32)	(±0.84)	(±0)	(±0.74)	(±0.79)	(±1.41)	(±2.20)	(±0.03)	(±0)	(±0.62)
th	31.73	73.87	16.71	75.23	61.64	49.31	38.92	66.08	49.14	26.13
11.42 μL/mL	(±0.87)	(±1.10)	(±3.13)	(±0.28)	(±1.70)	(±2.14)	(±3.77)	(±0.02)	(±0.32)	(±0.39)

**Table 4:** Inhibitory activity of the monofloral honeys, tested at 5.71 μL/mL and 11.42 μL/mL, on the adhesive capacity by five pathogenic bacteria. Results are expressed as percentage (average ± SD) and calculated assuming for the control (untreated bacteria) an inhibition value = zero. Legend: ca: cardoon honey; fir: fir honey; ivy: ivy honey; rhod: rhododendron honey; sul: sulla honey; sw: strawberry tree honey; th: tree of heaven honey. AB: *A. baumannii*; EC: *E. coli*; LM: *L. monocytogenes*; PS: *P. aeruginosa*; SA: *S. aureus.* MTT: data of the inhibitory action exhibited by the honey on bacterial metabolism, evaluated through the MTT test; CV: data of the inhibitory action exhibited by the honeys on the biofilm, evaluated through the Cristal violet assay.

	MTT					CV				
	AB	EC	LM	PS	SA	AB	EC	LM	PS	SA
ca	2.83	8.88	50.93	0	50.22	0	54.95	50.97	21.30	36.68
5.71 μL/mL	(±0.18)	(±1.53)	(±0.80)	(±0)	(±1.52)	(±0)	(±1.35)	(±0.66)	(±1.89)	(±0.75)
ca	44.97	43.48	53.73	15.73	55.21	19.95	52.20	68.05	37.30	55.14
11.42 μL/mL	(±1.25)	(±1.19)	(±1.52)	(±1.31)	(±2.71)	(±1.99)	(±5.45)	(±2.36)	(±0.80)	(±0.90)
fir	3.76	0	44.37	0	40.29	31.50	30.52	58.13	46.59	51.29
5.71 μL/mL	(±1.04)	(±0)	(±2.40)	(±0)	(±1.69)	(±1.77)	(±1.70)	(±0.77)	(±0.83)	(±1.73)
fir	40.53	49.16	60.98	41.37	56.81	33.69	43.06	68.30	54.85	58.97
11.42 μL/mL	(±2.43)	(±2.30)	(±3.10)	(±2.15)	(±1.03)	(±2.61)	(±1.54)	(±0.01)	(±1.79)	(±2.17)
ivy	0	0	34.65	0	47.18	22.33	13.97	52.07	26.00	32.97
5.71 μL/mL	(±0)	(±0)	(±1.49)	(±0)	(±0.83)	(±4.58)	(±2.16)	(±1.20)	(±1.52)	(±1.22)
ivy	0	0	55.86	0	49.81	32.80	40.99	70.34	41.22	51.76
11.42 μL/mL	(±0)	(±0)	(±0.86)	(±0)	(±0.83)	(±2.59)	(±0.62)	(±0.72)	(±2.81)	(±0.54)
rhod	0	0	13.25	0	38.65	49.83	47.92	36.56	31.60	24.98
5.71 μL/mL	(±0)	(±0)	(±1.50)	(±0)	(±1.05)	(±1.25)	(±0.64)	(±1.35)	(±0.53)	(±0.66)
rhod	7.16	0.11	28.30	0	47.71	51.67	66.63	44.30	36.67	27.54
11.42 μL/mL	(±1.51)	(±0.02)	(±1.18)	(±0)	(±1.00)	(±2.50)	(±0.19)	(±1.65)	(±0.75)	(±0.51)
sul	0	7.47	40.49	0	37.15	44.35	55.41	50.85	47.42	44.77
5.71 μL/mL	(±0)	(±1.38)	(±0.70)	(±0)	(±0.14)	(±3.05)	(±0.88)	(±0.89)	(±0.85)	(±1.24)
sul	13.48	22.19	42.37	0.57	45.38	51.77	66.24	71.57	52.74	49.19
11.42 μL/mL	(±2.27)	(±2.41)	(±0.39)	(±0.09)	(±1.10)	(±1.20)	(±1.50)	(±0.45)	(±0.70)	(±0.71)
sw	0	0	40.66	0	32.87	0	55.78	35.71	0	40.31
5.71 μL/mL	(±0)	(±0)	(±1.35)	(±0)	(±2.31)	(±0)	(±2.88)	(±0.79)	(±0)	(±1.18)
sw	2.18	7.76	42.52	0	39.82	8.42	56.64	47.47	18.91	43.81
11.42 μL/mL	(±0.94)	(±3.60)	(±0.56)	(±0)	(±1.17)	(±1.96)	(±1.00)	(±0.85)	(±1.54)	(±2.95)
th	0	4.46	28.27	0	37.50	33.52	59.39	64.33	34.81	79.46
5.71 μL/mL	(±0)	(±1.07)	(±0.91)	(±0)	(±1.04)	(±1.51)	(±1.49)	(±0.40)	(±1.27)	(±0.61)
th	23.37	25.52	46.78	0	44.42	34.87	73.66	69.49	61.56	84.27
11.42 μL/mL	(±1.23)	(±1.43)	(±0.96)	(±0)	(±0.81)	(±2.30)	(±1.96)	(±0.85)	(±1.73)	(±1.18)

**Table 5:** Inhibitory activity of the monofloral honeys, tested at 5.71 μL/mL and 11.42 μL/mL, on the inhibitory capacity on mature biofilm by five pathogenic bacteria. Results are expressed as percentage (average ±SD) and calculated assuming for the control (untreated bacteria) an inhibition value = zero. Legend: ca: cardoon honey; fir: fir honey; ivy: ivy honey; rhod: rhododendron honey; sul: sulla honey; sw: strawberry tree honey; th: tree of heaven honey. AB: *A. baumannii*; EC: *E. coli*; LM: *L. monocytogenes*; PS: *P. aeruginosa*; SA: *S. aureus.* MTT: data of the inhibitory action exhibited by the honey on bacterial metabolism, evaluated through the MTT test; CV: data of the inhibitory action exhibited by the honeys on the biofilm, evaluated through the Cristal violet assay.

	MTT					CV				
	AB	EC	LM	PS	SA	AB	EC	LM	PS	SA
ca	8.44	0	0	36.10	24.93	1.27	0	33.71	10.96	35.62
5.71 μL/mL	(±0.96)	(±0)	(±0)	(±2.74)	(±2.13)	(±0.74)	(±0)	(±0.88)	(±0.88)	(±1.42)
ca	13.97	0	0	11.66	41.06	8.66	0	53.84	19.94	40.33
11.42 μL/mL	(±1.75)	(±0)	(±0)	(±1.03)	(±1.76)	(±8.66)	(±0)	(±1.13)	(±0.28)	(±0.56)
fir	0	0	0	49.00	26.05	21.31	0	37.08	32.46	33.34
5.71 μL/mL	(±0)	(±0)	(±0)	(±1.38)	(±1.53)	(±1.07)	(±0)	(±1.61)	(±1.03)	(±0.98)
fir	85.19	0	42.40	70.76	44.29	23.95	0	52.43	34.41	38.21
11.42 μL/mL	(±1.39)	(±0)	(±3.83)	(±0.68)	(±2.70)	(±0.31)	(±0)	(±0.73)	(±0.50)	(±0.66)
ivy	0	0	44.48	30.84	25.17	6.70	0	33.86	42.14	44.59
5.71 μL/mL	(±0)	(±0)	(±1.49)	(±1.50)	(±1.07)	(±2.12)	(±0)	(±0.70)	(±1.37)	(±0.68)
ivy	6.66	0	53.80	38.05	43.89	22.30	0	50.98	44.54	64.96
11.42 μL/mL	(±0.70)	(±0)	(±2.01)	(±1.61)	(±2.62)	(±1.34)	(±0)	(±0.57)	(±1.72)	(±0.40)
rhod	41.57	0	0	0	0	10.62	0	31.83	20.22	32.38
5.71 μL/mL	(±0.68)	(±0)	(±0)	(±0)	(±0)	(±1.63)	(±0)	(±2.89)	(±0.87)	(±0.38)
rhod	45.27	0	23.16	37.66	1.10	16.58	0	44.83	28.13	34.77
11.42 μL/mL	(±1.15)	(±0)	(±2.33)	(±1.59)	(±0.09)	(±1.01)	(±0)	(±1.09)	(±0.54)	(±0.81)
sul	5.90	0	27.17	0	0	11.43	0	36.56	41.77	45.13
5.71 μL/mL	(±0.97)	(±0)	(±1.64)	(±0)	(±0)	(±2.23)	(±0)	(±0.32)	(±1.50)	(±0.69)
sul	31.28	0	36.45	19.50	46.23	23.04	0	37.47	44.98	48.15
11.42 μL/mL	(±1.65)	(±0)	(±2.49)	(±3.13)	(±1.17)	(±0.76)	(±0)	(±0.19)	(±0.70)	(±2.47)
sw	0	0	0	21.86	30.83	2.65	0	33.88	23.45	32.87
5.71 μL/mL	(±0)	(±0)	(±0)	(±1.58)	(±2.21)	(±0.31)	(±0)	(±0.49)	(±1.85)	(±0.52)
sw	9.02	0	0	28.81	55.47	11.87	0	54.10	51.08	37.24
11.42 μL/mL	(±1.72)	(±0)	(±0)	(±0.93)	(±3.53)	(±1.40)	(±0)	(±1.20)	(±1.10)	(±0.91)
th	18.78	0	0	27.40	37.84	23.54	0	11.84	25.70	46.85
5.71 μL/mL	(±3.06)	(±0)	(±0)	(±3.94)	(±1.86)	(±0.42)	(±0)	(±1.22)	(±0.57)	(±0.93)
th	57.95	0	34.28	66.98	43.78	27.51	0	30.41	26.89	76.64
11.42 μL/mL	(±0.71)	(±0)	(±4.91)	(±6.79)	(±0.64)	(±0.95)	(±0)	(±1.89)	(±0.45)	(±0.68)

## Data Availability

Not applicable.
